# 1(2,3),2(3,2),3(2,3),4(3,2)-Tetra­kis(1-benzothio­phena)cyclo­tetra­phane benzene sesquisolvate

**DOI:** 10.1107/S1600536809026178

**Published:** 2009-07-11

**Authors:** Zhi-Hua Wang, Jian-Wu Shi, Sheng Zhu, Hua Wang

**Affiliations:** a Key Laboratory for Special Functional Materials of the Ministry of Education, Henan University, Kaifeng 475004, People’s Republic of China

## Abstract

In the title compound, C_32_H_16_S_4_·1.5C_6_H_6_, the substituted cyclo­octa­tetra­ene (COT) ring compound has approximate local *D*_2_ point symmetry of the so-called ‘saddle’ form: the dihedral angles between neighboring benzo[*b*]thio­phene units are 61.33 (4), 61.61 (4), 56.443 (14) and 58.32 (4)°. The short distance [3.545 (1) Å] between an S atom and the centroid of a benzene ring in a neighboring mol­ecule may indicate S⋯π inter­actions in the crystal.

## Related literature

For the synthesis, see: Kauffmann *et al.* (1978 ▶). For related structures, see: Krömer *et al.* (2000 ▶); Mak & Wong (1987 ▶); Rajca *et al.* (1997 ▶, 2000 ▶); Wang *et al.* (2007 ▶).
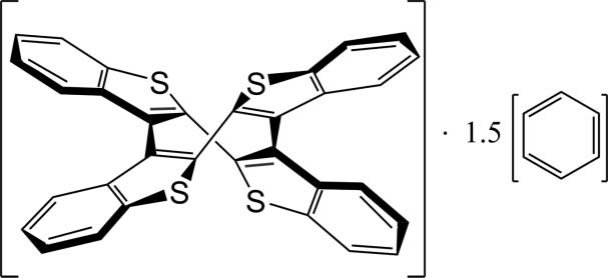


## Experimental

### 

#### Crystal data

C_32_H_16_S_4_·1.5C_6_H_6_
*M* *_r_* = 645.85
Triclinic, 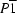

*a* = 9.5167 (10) Å
*b* = 13.3035 (14) Å
*c* = 13.9186 (15) Å
α = 65.674 (1)°
β = 84.646 (1)°
γ = 81.955 (1)°
*V* = 1588.7 (3) Å^3^
*Z* = 2
Mo *K*α radiation
μ = 0.33 mm^−1^
*T* = 294 K
0.41 × 0.25 × 0.15 mm

#### Data collection

Bruker SMART CCD diffractometer
Absorption correction: multi-scan (*SADABS*; Bruker, 2001 ▶) *T*_min_ = 0.877, *T*_max_ = 0.952
11927 measured reflections
5873 independent reflections
4670 reflections with *I* > 2σ(*I*)
*R*_int_ = 0.021

#### Refinement

*R*[*F* ^2^ > 2σ(*F* ^2^)] = 0.046
*wR*(*F* ^2^) = 0.121
*S* = 1.04
5873 reflections
394 parameters
H-atom parameters constrained
Δρ_max_ = 0.59 e Å^−3^
Δρ_min_ = −0.41 e Å^−3^

### 

Data collection: *SMART* (Bruker, 2001 ▶); cell refinement: *SAINT-Plus* (Bruker, 2001 ▶); data reduction: *SAINT-Plus*; program(s) used to solve structure: *SHELXS97* (Sheldrick, 2008 ▶); program(s) used to refine structure: *SHELXL97* (Sheldrick, 2008 ▶); molecular graphics: *SHELXTL* (Sheldrick, 2008 ▶); software used to prepare material for publication: *SHELXTL*.

## Supplementary Material

Crystal structure: contains datablocks global, I. DOI: 10.1107/S1600536809026178/hb2996sup1.cif

Structure factors: contains datablocks I. DOI: 10.1107/S1600536809026178/hb2996Isup2.hkl

Additional supplementary materials:  crystallographic information; 3D view; checkCIF report

## Figures and Tables

**Fig. 1.**
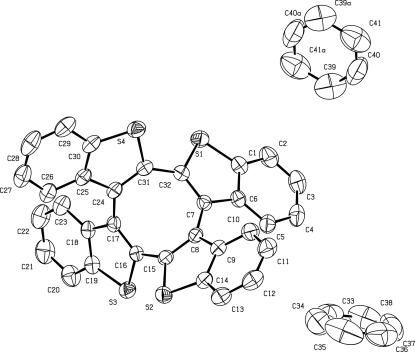
The molecular structure of (I) with displacement ellipsoids drawn at the 50% probability level.

**Fig. 2.**
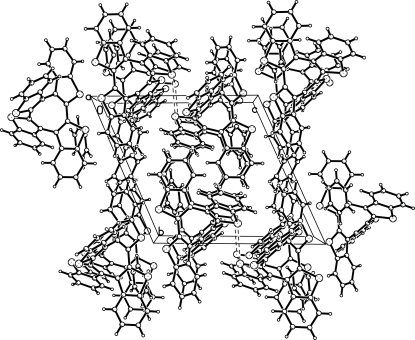
S-π interactions in crystal packing of (I).

## supplementary crystallographic information

### Comment

In current electronic and supramolecular chemistry, the rational design of macrocyclic compound represents one of the most exciting and rapidly developing fields, owing to their potential as functional materials (Krömer *et al.*, 2000). Mostly, the compounds with central COT ring have the "saddle" form (Rajca *et al.*, 1997, Mak & Wong, 1987, Rajca *et al.*, 2000, Wang *et al.*, 2007), such as tetra-*o*-phenylene, tetra-*o*-thiophene. In these "saddle" form molecules, the average dihedral angle of "saddle" form is different when the structural unit is different. Cycloocta[l,2-*b*:4,3-*b*':5,6-*b*":8,7-*b*"']tetrathionaphthene (I), with benza[*b*]thiophene as the structural unit was synthesized by Kauffmann *et al.* in 1978. In our ongoing studies of macrocyclic compounds, we find the structural unit plays an important role on the crystal structure and intermolecular interaction. In present paper, we report the crystal structure of I.

As shown in I (Fig. 1), three orthogonal C_2_ axes of symmetry are compatible with the D_2_ point group. One pair of the orthogonal chiral axes (*e.g.*, *R*,*R*) corresponds to the two 1,1'-linkages and the other pair (*e.g.*, S,*S*) corresponds to the two 2,2'-linkages of the benza[*b*]thiophene moieties. The four dihedral angles are 61.329 (35)° between C24—C25—C26—C27—C28—C29—C30—S4—C31 and C16—C17—C18—C23—C22—C21—C20—C19—S3, 61.610 (39)° between C8—C9—C10—C11—C12—C13—C14—S2—C15 and C1—C2—C3—C4—C5- C6—C7—C32—S1, 56.443 (14)° between C16—C17—C18—C23—C22—C21—C20—C19—S3 and C8—C9—C10—C11—C12—C13—C14—S2—C15, 58.315 (34)° between C24—C25—C26—C27—C28—C29—C30—S4—C31 and C1—C2—C3—C4—C5—C6—C7—C32—S1. The average value (59.4°) of the four dihedral angles is smaller than that in tetra-*o*-phenylene with 65° (Rajca *et al.*, 1997) and larger than that in tetra-*o*-thiophene with 51.7° (Wang *et al.*, 2007). The distance between S1 and the centroid of plane (C25, C26, C27, C28, C29, C30) is 3.378 Å, which indicating obvious S-π interaction between the neighboring molecules. Under the effect of S-π interaction, the molecular arranges with the reversal one in the crystal packing as shown in Fig. 2.

### Experimental

The title compound was synthesized according to the method of Kauffmann (1978). The overall yield was improved from 11% to 42.4%. To a solution of 3,3'-bibenzo[*b*]thiophene (0.3850 g, 1.45 mmol) in anhyd Et_2_O (50 ml), *n*-BuLi (2.28 M, 1.46 ml, 3.32 mmol, 2.3 eq) was added dropwise at -78 °C, then the reaction mixture was warmed slowly to 50 °C with refluxing for 2 h and then cooled to -78 °C. Dry CuCl_2_ (0.9741 g, 7.22 mmol, 5.0 equiv) was added at -78 °C and warmed slowly to -55 °C for 1 h, and then slowly warmed to ambient temperature overnight. After normal work-up, the crude product was purified by column chromatography on silica gel with PE (60–90 °C) /CHCl_3_ (3:1, *v*/*v*) as eluent to yield I (0.1621 g, 42.4%) as a white solid. mp>300 °C. ^1^H NMR (400 MHz, DMSO-*d*_6_): δ 8.12 (d, *J* = 8.0 Hz, 4H), 7.48 (t, *J* = 7.8 Hz, 4H), 7.37 (t, *J* = 7.4 Hz, 4H), 7.25 (d, *J* = 7.6 Hz, 4H). ^13^C NMR (100 MHz, CDCl_3_): δ 140.8, 138.6, 134.8, 131.3, 125.1, 124.7, 124.4, 122.3. IR: 3056, 2922 (C—H), 1435.5 (C=C) cm^-1^. HRMS (MAIDI-TOF MS EI^+^) m/*z* calcd for [C_32_H_16_S_4_] 528.0135, found 528.0131.  Yellow blocks of (I) were obtained by slow evaporation of benzene solution over a period of two weeks.

### Refinement

The H atoms were geometrically placed (C—H = 0.93Å) and refined as riding with *U*_iso_(H)=1.2*U*_eq_(C).

### Crystal data

**Table:**

C_32_H_16_S_4_·1.5C6H_6_	*Z* = 2
*M**_r_* = 645.85	*F*(000) = 670
Triclinic, *P*1	*D*_x_ = 1.350 Mg m^−^^3^
Hall symbol: -P 1	Mo *K*α radiation, λ = 0.71073 Å
*a* = 9.5167 (10) Å	Cell parameters from 3538 reflections
*b* = 13.3035 (14) Å	θ = 0.00–0.00°
*c* = 13.9186 (15) Å	µ = 0.33 mm^−^^1^
α = 65.674 (1)°	*T* = 294 K
β = 84.646 (1)°	Block, yellow
γ = 81.955 (1)°	0.41 × 0.25 × 0.15 mm
*V* = 1588.7 (3) Å^3^	

### Data collection

**Table:**

Bruker SMART CCD diffractometer	5873 independent reflections
Radiation source: fine-focus sealed tube	4670 reflections with *I* > 2σ(*I*)
graphite	*R*_int_ = 0.021
ω scans	θ_max_ = 25.5°, θ_min_ = 2.6°
Absorption correction: multi-scan (*SADABS*; Bruker, 2001)	*h* = −11→11
*T*_min_ = 0.877, *T*_max_ = 0.952	*k* = −16→16
11927 measured reflections	*l* = −16→16

### Refinement

**Table:**

Refinement on *F*^2^	Primary atom site location: structure-invariant direct methods
Least-squares matrix: full	Secondary atom site location: difference Fourier map
*R*[*F*^2^ > 2σ(*F*^2^)] = 0.046	Hydrogen site location: inferred from neighbouring sites
*wR*(*F*^2^) = 0.121	H-atom parameters constrained
*S* = 1.04	*w* = 1/[σ^2^(*F*_o_^2^) + (0.0507*P*)^2^ + 1.0002*P*] where *P* = (*F*_o_^2^ + 2*F*_c_^2^)/3
5873 reflections	(Δ/σ)_max_ = 0.001
394 parameters	Δρ_max_ = 0.59 e Å^−^^3^
0 restraints	Δρ_min_ = −0.41 e Å^−^^3^

### Special details

**Table:**

Geometry. All e.s.d.'s (except the e.s.d. in the dihedral angle between two l.s. planes)are estimated using the full covariance matrix. The cell e.s.d.'s are takeninto account individually in the estimation of e.s.d.'s in distances, anglesand torsion angles; correlations between e.s.d.'s in cell parameters are onlyused when they are defined by crystal symmetry. An approximate (isotropic)treatment of cell e.s.d.'s is used for estimating e.s.d.'s involving l.s. planes.
Refinement. Refinement of *F*^2^ against ALL reflections. The weighted *R*-factor *wR* and goodness of fit *S* are based on *F*^2^, conventional *R*-factors *R* are based on *F*, with *F* set to zero for negative *F*^2^. The threshold expression of *F*^2^ > σ(*F*^2^) is used only for calculating *R*-factors(gt) *etc*. and is not relevant to the choice of reflections for refinement. *R*-factors based on *F*^2^ are statistically about twice as large as those based on *F*, and *R*- factors based on ALL data will be even larger.

### Fractional atomic coordinates and isotropic or equivalent isotropic displacement parameters (Å^2^)

**Table:**

	*x*	*y*	*z*	*U*_iso_*/*U*_eq_	
C1	0.1068 (3)	0.6386 (2)	0.2230 (2)	0.0397 (6)	
C2	0.0961 (3)	0.5385 (2)	0.2153 (3)	0.0535 (7)	
H2	0.0810	0.5372	0.1507	0.064*	
C3	0.1082 (3)	0.4420 (2)	0.3052 (3)	0.0576 (8)	
H3	0.1015	0.3747	0.3013	0.069*	
C4	0.1304 (3)	0.4435 (2)	0.4019 (2)	0.0518 (7)	
H4	0.1388	0.3773	0.4617	0.062*	
C5	0.1400 (3)	0.5421 (2)	0.4101 (2)	0.0423 (6)	
H5	0.1547	0.5423	0.4751	0.051*	
C6	0.1276 (2)	0.6421 (2)	0.31980 (19)	0.0344 (5)	
C7	0.1259 (2)	0.75439 (19)	0.31116 (18)	0.0319 (5)	
C8	0.1456 (2)	0.78278 (18)	0.40068 (18)	0.0321 (5)	
C9	0.2687 (2)	0.74267 (19)	0.46507 (18)	0.0347 (5)	
C10	0.3936 (3)	0.6788 (2)	0.4529 (2)	0.0440 (6)	
H10	0.4040	0.6542	0.3988	0.053*	
C11	0.5009 (3)	0.6526 (2)	0.5222 (2)	0.0521 (7)	
H11	0.5836	0.6094	0.5150	0.063*	
C12	0.4870 (3)	0.6902 (2)	0.6029 (2)	0.0524 (7)	
H12	0.5608	0.6719	0.6486	0.063*	
C13	0.3664 (3)	0.7535 (2)	0.6163 (2)	0.0475 (7)	
H13	0.3576	0.7785	0.6700	0.057*	
C14	0.2573 (3)	0.7792 (2)	0.54677 (19)	0.0380 (6)	
C15	0.0472 (3)	0.84729 (19)	0.43395 (18)	0.0332 (5)	
C16	−0.0901 (2)	0.90222 (18)	0.38884 (18)	0.0323 (5)	
C17	−0.1138 (2)	0.97723 (18)	0.28804 (18)	0.0326 (5)	
C18	−0.2632 (3)	1.01481 (19)	0.27136 (19)	0.0358 (5)	
C19	−0.3476 (3)	0.9663 (2)	0.3631 (2)	0.0398 (6)	
C20	−0.4957 (3)	0.9883 (2)	0.3643 (3)	0.0529 (7)	
H20	−0.5503	0.9551	0.4256	0.063*	
C21	−0.5581 (3)	1.0605 (3)	0.2721 (3)	0.0611 (8)	
H21	−0.6563	1.0774	0.2714	0.073*	
C22	−0.4768 (3)	1.1085 (3)	0.1800 (3)	0.0592 (8)	
H22	−0.5216	1.1562	0.1184	0.071*	
C23	−0.3308 (3)	1.0868 (2)	0.1784 (2)	0.0469 (6)	
H23	−0.2777	1.1197	0.1163	0.056*	
C24	0.0004 (2)	1.01805 (19)	0.20673 (18)	0.0338 (5)	
C25	0.0249 (3)	1.1333 (2)	0.15376 (18)	0.0362 (5)	
C26	−0.0462 (3)	1.2254 (2)	0.1694 (2)	0.0448 (6)	
H26	−0.1216	1.2167	0.2187	0.054*	
C27	−0.0033 (4)	1.3285 (2)	0.1110 (2)	0.0567 (8)	
H27	−0.0507	1.3899	0.1208	0.068*	
C28	0.1104 (4)	1.3430 (2)	0.0372 (3)	0.0626 (9)	
H28	0.1383	1.4135	−0.0007	0.075*	
C29	0.1816 (3)	1.2545 (2)	0.0197 (2)	0.0532 (7)	
H29	0.2564	1.2643	−0.0301	0.064*	
C30	0.1387 (3)	1.1492 (2)	0.07885 (19)	0.0407 (6)	
C31	0.0942 (3)	0.9515 (2)	0.17253 (18)	0.0355 (5)	
C32	0.1042 (2)	0.83069 (19)	0.21043 (18)	0.0339 (5)	
C33	0.2633 (3)	0.3588 (4)	0.7185 (2)	0.1020 (15)	
H33	0.1831	0.3940	0.6795	0.122*	
C34	0.3502 (5)	0.4197 (2)	0.7428 (3)	0.1141 (19)	
H34	0.3282	0.4961	0.7200	0.137*	
C35	0.4697 (4)	0.3670 (4)	0.8010 (3)	0.118 (2)	
H35	0.5277	0.4081	0.8172	0.142*	
C36	0.5032 (3)	0.2531 (4)	0.8351 (2)	0.1134 (18)	
H36	0.5837	0.2179	0.8737	0.136*	
C37	0.4159 (5)	0.1920 (2)	0.8112 (3)	0.1136 (18)	
H37	0.4373	0.1155	0.8345	0.136*	
C38	0.2969 (4)	0.2450 (4)	0.7526 (3)	0.1034 (16)	
H38	0.2391	0.2039	0.7360	0.124*	
C39	0.4111 (5)	0.4474 (5)	0.0892 (4)	0.1025 (15)	
H39	0.3517	0.4117	0.1468	0.123*	
C40	0.5457 (6)	0.4043 (3)	0.0777 (4)	0.0949 (14)	
H40	0.5791	0.3391	0.1330	0.114*	
C41	0.6349 (5)	0.4453 (5)	−0.0039 (5)	0.1049 (15)	
H41	0.7238	0.4075	−0.0073	0.126*	
S1	0.08749 (7)	0.77085 (6)	0.12239 (5)	0.04378 (18)	
S2	0.09726 (7)	0.86121 (6)	0.54491 (5)	0.04315 (18)	
S3	−0.24494 (7)	0.87555 (5)	0.46789 (5)	0.04184 (18)	
S4	0.21218 (7)	1.02532 (6)	0.07232 (5)	0.04525 (18)	

### Atomic displacement parameters (Å^2^)

**Table:**

	*U*^11^	*U*^22^	*U*^33^	*U*^12^	*U*^13^	*U*^23^
C1	0.0360 (13)	0.0424 (14)	0.0456 (15)	−0.0014 (11)	−0.0032 (11)	−0.0235 (12)
C2	0.0588 (18)	0.0521 (18)	0.0627 (19)	−0.0048 (14)	−0.0055 (15)	−0.0361 (16)
C3	0.0627 (19)	0.0404 (16)	0.079 (2)	−0.0048 (14)	−0.0038 (16)	−0.0334 (16)
C4	0.0505 (17)	0.0332 (14)	0.0655 (19)	−0.0007 (12)	−0.0030 (14)	−0.0150 (13)
C5	0.0404 (14)	0.0366 (14)	0.0457 (15)	0.0000 (11)	−0.0030 (11)	−0.0136 (12)
C6	0.0302 (12)	0.0353 (13)	0.0400 (13)	−0.0012 (10)	−0.0030 (10)	−0.0180 (11)
C7	0.0289 (12)	0.0340 (12)	0.0335 (12)	0.0007 (9)	−0.0036 (9)	−0.0150 (10)
C8	0.0369 (13)	0.0277 (12)	0.0294 (12)	−0.0030 (10)	−0.0037 (10)	−0.0089 (10)
C9	0.0361 (13)	0.0332 (13)	0.0313 (12)	−0.0042 (10)	−0.0038 (10)	−0.0088 (10)
C10	0.0386 (14)	0.0479 (15)	0.0423 (15)	0.0001 (12)	−0.0018 (11)	−0.0167 (12)
C11	0.0365 (15)	0.0588 (18)	0.0518 (17)	0.0031 (13)	−0.0085 (12)	−0.0142 (14)
C12	0.0424 (16)	0.0625 (19)	0.0447 (16)	−0.0080 (14)	−0.0149 (12)	−0.0107 (14)
C13	0.0498 (16)	0.0538 (17)	0.0402 (15)	−0.0083 (13)	−0.0102 (12)	−0.0179 (13)
C14	0.0419 (14)	0.0351 (13)	0.0342 (13)	−0.0050 (11)	−0.0045 (11)	−0.0104 (11)
C15	0.0394 (13)	0.0300 (12)	0.0282 (12)	−0.0045 (10)	−0.0028 (10)	−0.0093 (10)
C16	0.0365 (13)	0.0274 (12)	0.0329 (12)	−0.0015 (10)	−0.0015 (10)	−0.0128 (10)
C17	0.0367 (13)	0.0256 (12)	0.0359 (13)	−0.0017 (10)	−0.0040 (10)	−0.0129 (10)
C18	0.0377 (13)	0.0281 (12)	0.0420 (14)	−0.0016 (10)	−0.0044 (11)	−0.0146 (11)
C19	0.0383 (14)	0.0335 (13)	0.0470 (15)	−0.0005 (11)	−0.0032 (11)	−0.0164 (12)
C20	0.0386 (15)	0.0533 (17)	0.0676 (19)	−0.0058 (13)	0.0030 (14)	−0.0260 (15)
C21	0.0336 (15)	0.064 (2)	0.082 (2)	0.0037 (14)	−0.0114 (15)	−0.0259 (18)
C22	0.0475 (17)	0.0550 (18)	0.068 (2)	0.0022 (14)	−0.0223 (15)	−0.0157 (16)
C23	0.0462 (16)	0.0415 (15)	0.0473 (16)	−0.0028 (12)	−0.0099 (12)	−0.0113 (12)
C24	0.0354 (13)	0.0314 (12)	0.0315 (12)	−0.0040 (10)	−0.0074 (10)	−0.0082 (10)
C25	0.0399 (13)	0.0333 (13)	0.0312 (12)	−0.0045 (10)	−0.0107 (10)	−0.0069 (10)
C26	0.0528 (16)	0.0352 (14)	0.0433 (15)	−0.0032 (12)	−0.0115 (12)	−0.0113 (12)
C27	0.074 (2)	0.0338 (15)	0.0590 (19)	−0.0044 (14)	−0.0219 (16)	−0.0124 (14)
C28	0.073 (2)	0.0376 (16)	0.065 (2)	−0.0198 (15)	−0.0213 (17)	−0.0003 (15)
C29	0.0536 (17)	0.0489 (17)	0.0443 (16)	−0.0188 (14)	−0.0070 (13)	−0.0010 (13)
C30	0.0433 (14)	0.0392 (14)	0.0325 (13)	−0.0090 (11)	−0.0094 (11)	−0.0043 (11)
C31	0.0373 (13)	0.0363 (13)	0.0301 (12)	−0.0049 (10)	−0.0038 (10)	−0.0100 (10)
C32	0.0329 (12)	0.0363 (13)	0.0342 (13)	−0.0038 (10)	−0.0003 (10)	−0.0160 (11)
C33	0.073 (3)	0.145 (5)	0.067 (3)	−0.004 (3)	0.014 (2)	−0.028 (3)
C34	0.137 (5)	0.065 (3)	0.125 (4)	−0.030 (3)	0.063 (4)	−0.032 (3)
C35	0.098 (4)	0.177 (6)	0.137 (5)	−0.086 (4)	0.048 (3)	−0.111 (5)
C36	0.057 (3)	0.182 (6)	0.090 (3)	0.013 (3)	−0.001 (2)	−0.053 (4)
C37	0.129 (4)	0.073 (3)	0.131 (4)	−0.004 (3)	0.050 (4)	−0.047 (3)
C38	0.099 (4)	0.139 (5)	0.111 (4)	−0.058 (3)	0.036 (3)	−0.084 (4)
C39	0.089 (3)	0.132 (4)	0.089 (3)	−0.032 (3)	−0.004 (3)	−0.040 (3)
C40	0.139 (4)	0.054 (2)	0.079 (3)	0.003 (3)	−0.039 (3)	−0.010 (2)
C41	0.067 (3)	0.154 (5)	0.115 (4)	0.001 (3)	−0.006 (3)	−0.079 (4)
S1	0.0533 (4)	0.0462 (4)	0.0358 (3)	−0.0035 (3)	−0.0077 (3)	−0.0201 (3)
S2	0.0516 (4)	0.0441 (4)	0.0374 (4)	0.0043 (3)	−0.0097 (3)	−0.0215 (3)
S3	0.0403 (4)	0.0389 (4)	0.0388 (4)	−0.0031 (3)	0.0036 (3)	−0.0097 (3)
S4	0.0438 (4)	0.0461 (4)	0.0380 (4)	−0.0069 (3)	0.0046 (3)	−0.0099 (3)

### Geometric parameters (Å, °)

**Table:**

C1—C2	1.399 (4)	C21—H21	0.9300
C1—C6	1.400 (3)	C22—C23	1.379 (4)
C1—S1	1.737 (3)	C22—H22	0.9300
C2—C3	1.374 (4)	C23—H23	0.9300
C2—H2	0.9300	C24—C31	1.362 (3)
C3—C4	1.390 (4)	C24—C25	1.444 (3)
C3—H3	0.9300	C25—C26	1.403 (4)
C4—C5	1.379 (4)	C25—C30	1.405 (4)
C4—H4	0.9300	C26—C27	1.374 (4)
C5—C6	1.403 (3)	C26—H26	0.9300
C5—H5	0.9300	C27—C28	1.397 (5)
C6—C7	1.446 (3)	C27—H27	0.9300
C7—C32	1.364 (3)	C28—C29	1.373 (4)
C7—C8	1.477 (3)	C28—H28	0.9300
C8—C15	1.360 (3)	C29—C30	1.398 (4)
C8—C9	1.445 (3)	C29—H29	0.9300
C9—C14	1.400 (3)	C30—S4	1.733 (3)
C9—C10	1.400 (3)	C31—C32	1.462 (3)
C10—C11	1.379 (4)	C31—S4	1.749 (2)
C10—H10	0.9300	C32—S1	1.743 (2)
C11—C12	1.395 (4)	C33—C38	1.3855
C11—H11	0.9300	C33—C34	1.3860
C12—C13	1.370 (4)	C33—H33	0.9300
C12—H12	0.9300	C34—C35	1.3869
C13—C14	1.395 (3)	C34—H34	0.9300
C13—H13	0.9300	C35—C36	1.3868
C14—S2	1.741 (3)	C35—H35	0.9300
C15—C16	1.464 (3)	C36—C37	1.3868
C15—S2	1.745 (2)	C36—H36	0.9300
C16—C17	1.363 (3)	C37—C38	1.3862
C16—S3	1.741 (2)	C37—H37	0.9300
C17—C18	1.447 (3)	C38—H38	0.9300
C17—C24	1.478 (3)	C39—C40	1.352 (6)
C18—C19	1.402 (3)	C39—C41^i^	1.471 (7)
C18—C23	1.402 (3)	C39—H39	0.9300
C19—C20	1.399 (4)	C40—C41	1.322 (6)
C19—S3	1.739 (3)	C40—H40	0.9300
C20—C21	1.374 (4)	C41—C39^i^	1.471 (7)
C20—H20	0.9300	C41—H41	0.9300
C21—C22	1.388 (4)		
			
C2—C1—C6	121.4 (3)	C23—C22—H22	119.4
C2—C1—S1	126.9 (2)	C21—C22—H22	119.4
C6—C1—S1	111.54 (18)	C22—C23—C18	119.5 (3)
C3—C2—C1	118.5 (3)	C22—C23—H23	120.3
C3—C2—H2	120.8	C18—C23—H23	120.3
C1—C2—H2	120.8	C31—C24—C25	112.1 (2)
C2—C3—C4	121.0 (3)	C31—C24—C17	123.9 (2)
C2—C3—H3	119.5	C25—C24—C17	124.0 (2)
C4—C3—H3	119.5	C26—C25—C30	118.9 (2)
C5—C4—C3	120.8 (3)	C26—C25—C24	128.9 (2)
C5—C4—H4	119.6	C30—C25—C24	112.2 (2)
C3—C4—H4	119.6	C27—C26—C25	119.2 (3)
C4—C5—C6	119.7 (3)	C27—C26—H26	120.4
C4—C5—H5	120.2	C25—C26—H26	120.4
C6—C5—H5	120.2	C26—C27—C28	121.1 (3)
C1—C6—C5	118.6 (2)	C26—C27—H27	119.4
C1—C6—C7	112.3 (2)	C28—C27—H27	119.4
C5—C6—C7	129.0 (2)	C29—C28—C27	120.9 (3)
C32—C7—C6	111.9 (2)	C29—C28—H28	119.5
C32—C7—C8	124.2 (2)	C27—C28—H28	119.5
C6—C7—C8	124.0 (2)	C28—C29—C30	118.3 (3)
C15—C8—C9	112.2 (2)	C28—C29—H29	120.9
C15—C8—C7	123.5 (2)	C30—C29—H29	120.9
C9—C8—C7	124.2 (2)	C29—C30—C25	121.5 (3)
C14—C9—C10	118.8 (2)	C29—C30—S4	126.9 (2)
C14—C9—C8	112.2 (2)	C25—C30—S4	111.60 (18)
C10—C9—C8	128.9 (2)	C24—C31—C32	127.3 (2)
C11—C10—C9	119.3 (3)	C24—C31—S4	112.96 (18)
C11—C10—H10	120.4	C32—C31—S4	119.71 (18)
C9—C10—H10	120.4	C7—C32—C31	127.2 (2)
C10—C11—C12	120.8 (3)	C7—C32—S1	113.15 (18)
C10—C11—H11	119.6	C31—C32—S1	119.69 (17)
C12—C11—H11	119.6	C38—C33—C34	119.6
C13—C12—C11	121.2 (3)	C38—C33—H33	120.2
C13—C12—H12	119.4	C34—C33—H33	120.2
C11—C12—H12	119.4	C33—C34—C35	120.1
C12—C13—C14	118.1 (3)	C33—C34—H34	119.9
C12—C13—H13	121.0	C35—C34—H34	119.9
C14—C13—H13	121.0	C36—C35—C34	120.2
C13—C14—C9	121.8 (2)	C36—C35—H35	119.9
C13—C14—S2	126.7 (2)	C34—C35—H35	119.9
C9—C14—S2	111.44 (18)	C35—C36—C37	119.7
C8—C15—C16	127.9 (2)	C35—C36—H36	120.2
C8—C15—S2	113.03 (18)	C37—C36—H36	120.2
C16—C15—S2	119.06 (17)	C38—C37—C36	120.0
C17—C16—C15	127.1 (2)	C38—C37—H37	120.0
C17—C16—S3	113.18 (18)	C36—C37—H37	120.0
C15—C16—S3	119.76 (17)	C33—C38—C37	120.4
C16—C17—C18	111.9 (2)	C33—C38—H38	119.8
C16—C17—C24	123.9 (2)	C37—C38—H38	119.8
C18—C17—C24	124.2 (2)	C40—C39—C41^i^	115.2 (4)
C19—C18—C23	118.4 (2)	C40—C39—H39	122.4
C19—C18—C17	112.2 (2)	C41^i^—C39—H39	122.4
C23—C18—C17	129.3 (2)	C41—C40—C39	126.8 (4)
C20—C19—C18	122.0 (2)	C41—C40—H40	116.6
C20—C19—S3	126.6 (2)	C39—C40—H40	116.6
C18—C19—S3	111.39 (18)	C40—C41—C39^i^	117.8 (4)
C21—C20—C19	118.0 (3)	C40—C41—H41	121.1
C21—C20—H20	121.0	C39^i^—C41—H41	121.1
C19—C20—H20	121.0	C1—S1—C32	91.15 (12)
C20—C21—C22	121.0 (3)	C14—S2—C15	91.10 (12)
C20—C21—H21	119.5	C19—S3—C16	91.25 (12)
C22—C21—H21	119.5	C30—S4—C31	91.14 (12)
C23—C22—C21	121.1 (3)		
			
C6—C1—C2—C3	−0.8 (4)	C21—C22—C23—C18	0.1 (4)
S1—C1—C2—C3	−177.0 (2)	C19—C18—C23—C22	0.6 (4)
C1—C2—C3—C4	0.1 (4)	C17—C18—C23—C22	177.9 (3)
C2—C3—C4—C5	0.3 (5)	C16—C17—C24—C31	60.4 (3)
C3—C4—C5—C6	−0.1 (4)	C18—C17—C24—C31	−121.9 (3)
C2—C1—C6—C5	1.0 (4)	C16—C17—C24—C25	−120.1 (3)
S1—C1—C6—C5	177.75 (18)	C18—C17—C24—C25	57.7 (3)
C2—C1—C6—C7	−176.0 (2)	C31—C24—C25—C26	−178.0 (2)
S1—C1—C6—C7	0.7 (3)	C17—C24—C25—C26	2.4 (4)
C4—C5—C6—C1	−0.5 (4)	C31—C24—C25—C30	0.6 (3)
C4—C5—C6—C7	175.9 (2)	C17—C24—C25—C30	−179.0 (2)
C1—C6—C7—C32	0.0 (3)	C30—C25—C26—C27	0.2 (4)
C5—C6—C7—C32	−176.6 (2)	C24—C25—C26—C27	178.7 (2)
C1—C6—C7—C8	−179.8 (2)	C25—C26—C27—C28	−0.5 (4)
C5—C6—C7—C8	3.6 (4)	C26—C27—C28—C29	0.8 (5)
C32—C7—C8—C15	59.8 (3)	C27—C28—C29—C30	−0.9 (4)
C6—C7—C8—C15	−120.5 (3)	C28—C29—C30—C25	0.6 (4)
C32—C7—C8—C9	−122.4 (3)	C28—C29—C30—S4	−179.3 (2)
C6—C7—C8—C9	57.4 (3)	C26—C25—C30—C29	−0.3 (4)
C15—C8—C9—C14	0.3 (3)	C24—C25—C30—C29	−179.0 (2)
C7—C8—C9—C14	−177.8 (2)	C26—C25—C30—S4	179.67 (19)
C15—C8—C9—C10	−176.9 (2)	C24—C25—C30—S4	0.9 (3)
C7—C8—C9—C10	5.0 (4)	C25—C24—C31—C32	178.0 (2)
C14—C9—C10—C11	0.7 (4)	C17—C24—C31—C32	−2.5 (4)
C8—C9—C10—C11	177.8 (2)	C25—C24—C31—S4	−1.9 (3)
C9—C10—C11—C12	−0.8 (4)	C17—C24—C31—S4	177.73 (18)
C10—C11—C12—C13	0.4 (4)	C6—C7—C32—C31	179.1 (2)
C11—C12—C13—C14	0.2 (4)	C8—C7—C32—C31	−1.1 (4)
C12—C13—C14—C9	−0.3 (4)	C6—C7—C32—S1	−0.7 (3)
C12—C13—C14—S2	−177.9 (2)	C8—C7—C32—S1	179.04 (18)
C10—C9—C14—C13	−0.2 (4)	C24—C31—C32—C7	−58.1 (4)
C8—C9—C14—C13	−177.7 (2)	S4—C31—C32—C7	121.7 (2)
C10—C9—C14—S2	177.79 (19)	C24—C31—C32—S1	121.7 (2)
C8—C9—C14—S2	0.2 (3)	S4—C31—C32—S1	−58.5 (2)
C9—C8—C15—C16	−180.0 (2)	C38—C33—C34—C35	−0.1
C7—C8—C15—C16	−1.9 (4)	C33—C34—C35—C36	0.1
C9—C8—C15—S2	−0.7 (3)	C34—C35—C36—C37	−0.4
C7—C8—C15—S2	177.35 (18)	C35—C36—C37—C38	0.7
C8—C15—C16—C17	−58.0 (4)	C34—C33—C38—C37	0.4
S2—C15—C16—C17	122.8 (2)	C36—C37—C38—C33	−0.7
C8—C15—C16—S3	123.5 (2)	C41^i^—C39—C40—C41	−4.3 (8)
S2—C15—C16—S3	−55.7 (2)	C39—C40—C41—C39^i^	4.4 (8)
C15—C16—C17—C18	−179.7 (2)	C2—C1—S1—C32	175.6 (3)
S3—C16—C17—C18	−1.0 (3)	C6—C1—S1—C32	−0.98 (19)
C15—C16—C17—C24	−1.7 (4)	C7—C32—S1—C1	1.00 (19)
S3—C16—C17—C24	177.01 (18)	C31—C32—S1—C1	−178.9 (2)
C16—C17—C18—C19	0.8 (3)	C13—C14—S2—C15	177.3 (2)
C24—C17—C18—C19	−177.2 (2)	C9—C14—S2—C15	−0.55 (19)
C16—C17—C18—C23	−176.6 (2)	C8—C15—S2—C14	0.74 (19)
C24—C17—C18—C23	5.4 (4)	C16—C15—S2—C14	−179.94 (19)
C23—C18—C19—C20	−0.4 (4)	C20—C19—S3—C16	177.5 (2)
C17—C18—C19—C20	−178.1 (2)	C18—C19—S3—C16	−0.24 (19)
C23—C18—C19—S3	177.46 (19)	C17—C16—S3—C19	0.72 (19)
C17—C18—C19—S3	−0.3 (3)	C15—C16—S3—C19	179.49 (19)
C18—C19—C20—C21	−0.4 (4)	C29—C30—S4—C31	178.3 (2)
S3—C19—C20—C21	−178.0 (2)	C25—C30—S4—C31	−1.62 (19)
C19—C20—C21—C22	1.2 (5)	C24—C31—S4—C30	2.02 (19)
C20—C21—C22—C23	−1.0 (5)	C32—C31—S4—C30	−177.8 (2)

Symmetry codes: (i) −*x*+1, −*y*+1, −*z*.

## References

[bb1] Bruker (2001). *SAINT-Plus*, *SMART* and *SADABS* Bruker AXS Inc., Madison, Wisconsin, USA.

[bb2] Kauffmann, T., Greving, B., Kriegesmann, R., Mitschker, A. & Woltermann, A. (1978). *Chem. Ber.* **111**, 1330–1336.

[bb3] Krömer, J., Rios-Carreras, I., Fuhrmann, G., Musch, C., Wunderlin, M., Debaerdemaeker, T., Mena-Osteritz, E. & Bäuerle, P. (2000). *Angew. Chem. Int. Ed. Engl.* **39**, 3481—3486. 11091396

[bb4] Mak, T. C. W. & Wong, H. N. C. (1987). *Top. Curr. Chem.* **140**, 141–164.

[bb5] Rajca, A., Safronov, A., Rajca, S. & Shoemaker, R. (1997). *Angew. Chem. Int. Ed. Engl.* **36**, 488–491.

[bb6] Rajca, A., Safronov, A., Rajca, S. & Wongsriratanakul, J. (2000). *J. Am. Chem. Soc.* **122**, 3351–3357.

[bb7] Sheldrick, G. M. (2008). *Acta Cryst.* A**64**, 112–122. 10.1107/S0108767307043930 18156677

[bb9] Wang, Y., Wang, Z., Zhao, D., Wang, Z., Chen, Y. & Wang, H. (2007). *Synlett*, pp. 2390–2394.

